# Whole genome assessment of the retinal response to diabetes reveals a progressive neurovascular inflammatory response

**DOI:** 10.1186/1755-8794-1-26

**Published:** 2008-06-13

**Authors:** Robert M Brucklacher, Kruti M Patel, Heather D VanGuilder, Georgina V Bixler, Alistair J Barber, David A Antonetti, Cheng-Mao Lin, Kathryn F LaNoue, Thomas W Gardner, Sarah K Bronson, Willard M Freeman

**Affiliations:** 1 Functional Genomics Core Facility, Penn State College of Medicine, Hershey, Pennsylvania, USA; 2 Department of Ophthalmology, Penn State College of Medicine, Hershey, Pennsylvania, USA; 3 Department of Cellular & Molecular Physiology, Penn State College of Medicine, Hershey, Pennsylvania, USA; 4 Department of Pharmacology, Penn State College of Medicine, Hershey, Pennsylvania, USA

## Abstract

**Background:**

Despite advances in the understanding of diabetic retinopathy, the nature and time course of molecular changes in the retina with diabetes are incompletely described. This study characterized the functional and molecular phenotype of the retina with increasing durations of diabetes.

**Results:**

Using the streptozotocin-induced rat model of diabetes, levels of retinal permeability, caspase activity, and gene expression were examined after 1 and 3 months of diabetes. Gene expression changes were identified by whole genome microarray and confirmed by qPCR in the same set of animals as used in the microarray analyses and subsequently validated in independent sets of animals. Increased levels of vascular permeability and caspase-3 activity were observed at 3 months of diabetes, but not 1 month. Significantly more and larger magnitude gene expression changes were observed after 3 months than after 1 month of diabetes. Quantitative PCR validation of selected genes related to inflammation, microvasculature and neuronal function confirmed gene expression changes in multiple independent sets of animals.

**Conclusion:**

These changes in permeability, apoptosis, and gene expression provide further evidence of progressive retinal malfunction with increasing duration of diabetes. The specific gene expression changes confirmed in multiple sets of animals indicate that pro-inflammatory, anti-vascular barrier, and neurodegenerative changes occur in tandem with functional increases in apoptosis and vascular permeability. These responses are shared with the clinically documented inflammatory response in diabetic retinopathy suggesting that this model may be used to test anti-inflammatory therapeutics.

## Background

Diabetic retinopathy (DR) is one of the most debilitating diabetic complications. DR is best prevented by intensive glycemic control as demonstrated by the Diabetes Control and Complications Trial (DCCT) study [1]. Established treatments of DR are limited to panretinal laser photocoagulation therapy and control of hypertension [2].

However, the ability to achieve intensive glycemic control through insulin is limited by hypoglycemia, and given that islet cell transplants are not widely available, pharmacotherapies for prevention and treatment of DR are needed. The PKC inhibitor ruboxistaurin has shown promise as a therapeutic agent [3], but this compound did not receive FDA approval. Therefore, continued development is needed for DR therapies.

The etiology and pathological processes of DR remain imperfectly understood with the major pathophysiolgical hypotheses being microvascular proliferation and/or permeability [4], advanced glycation endproducts (AGEs) [5], PKC activation [6], oxidative stress [7], inflammation [8], loss of insulin signaling, and neurodegeneration [9]. Previously, we proposed that these different processes are not mutually exclusive, but rather could represent different components of a feed-forward cycle of nutrient overload leading to chronic inflammation, neurodegeneration, and compromised blood-retinal barrier function [9].

Previous work has provided important insights into the genomic and proteomic changes associated with DR [10-13]. This study sought to tie changes in retinal gene expression to functional changes in the retina as measured through vascular permeability and apoptosis. Clinical DR is a progressive disease; therefore the rodent model should also demonstrate progressive dysfunction with increasing duration of diabetes. This study accomplished two goals in examining aspects of the retinal response to diabetes: first, to demonstrate functional changes in retinal permeability and caspase-3 activity (as a surrogate for apoptosis) after 1 and 3 months of experimental diabetes, and second, to identify and confirm retinal gene expression alterations at 1 and 3 months of diabetes.

## Results

At harvest, as predicted by earlier assessments, diabetic rats were hyperglycemic and underweight in comparison to control rats (Table 1). These data and the methods described are representative of experiments performed by the Penn State JDRF Animal Models Core since 2003 [14].

**Table 1:** Animal Data. Mean weight and blood glucose at the time of sacrifice for the different sets of animals and their respective analyses. Mean ± S.E.M.

			**N**	**Weight (g)**	**Blood Glucose (mg/dL)**	**Analyses**
**Set 1**	**1 Month**	**Control**	5	411 ± 7	87 ± 3	Microarray & qPCR
		**STZ**	6	317 ± 16	303 ± 8	
	**3 Month**	**Control**	6	617 ± 11	93 ± 4	Microarray & qPCR
		**STZ**	6	303 ± 8	370 ± 21	
**Set 2**	**1 Month**	**Control**	6	391 ± 9	114 ± 12	qPCR
		**STZ**	6	264 ± 16	321 ± 16	
	**3 Month**	**Control**	6	587 ± 18	106 ± 3	qPCR
		**STZ**	6	319 ± 16	335 ± 10	
**Set 3**	**3 Month**	**Control**	6	574 ± 8	91 ± 13	qPCR
		**STZ**	8	364 ± 17	377 ± 33	
**Set 4**	**1 Month**	**Control**	8	379 ± 11	90 ± 3	Caspase
		**STZ**	8	277 ± 9	426 ± 23	
	**3 Month**	**Control**	8	545 ± 22	78 ± 2	Caspase
		**STZ**	7	341 ± 27	475 ± 24	
**Set 5**	**1 Month**	**Control**	9	379 ± 11	133 ± 12	Permeability
		**STZ**	11	296 ± 12	486 ± 21	
	**3 Month**	**Control**	8	592 ± 10	116 ± 4	Permeability
		**STZ**	7	325 ± 23	430 ± 37	

### Vascular permeability and apoptosis

Retinal vascular permeability, as measured by FITC-BSA incorporation into nonvascular retinal tissue, was unchanged at 1 month of diabetes but was significantly increased (52%, p < 0.05) after 3 months of diabetes (Table 2). Similarly, caspase-3 activity was unchanged after 1 month of diabetes but was increased significantly after 3 months of diabetes (24%, p < 0.05) (Table 2).

**Table 2:** Caspase and Permeability Data: Retinal vascular permeability and apoptosis was measured at 1 and 3 month time points.

			**N**	**Percent Mean Control**
**Permeability**	**1 Month**	**Control**	9	100 ± 8%
		**STZ**	11	98 ± 8%
	**3 Month**	**Control**	8	100 ± 3%
		**STZ**	7	152 ± 8%*
**Caspase-3 Activity**	**1 Month**	**Control**	8	100 ± 9%
		**STZ**	8	100 ± 10%
	**3 Month**	**Control**	8	100 ± 17%
		**STZ**	7	125 ± 16%*

Values are expressed as a percentage of the control condition at that time point ± S.E.M. *p < 0.05, t-test.

### Microarray analysis

**Figure 1:**
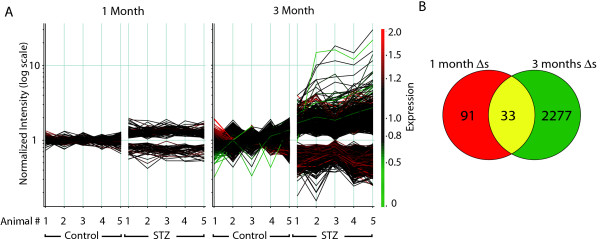
**Microarray analysis**. **A**. Differences in RNA expression 1 or 3 months after STZ induced diabetes were analyzed by Codelink whole genome microarray. After filtering for genes detected as present, differential expression was determined by ANOVA p < 0.01 and a fold change of 1.2 fold or greater. For differentially expressed genes values for each of the five animals in control and STZ treated groups are given in log scale normalized as described in the Methods. Lines are colored according to their relative normalized expression level. Far more changes and larger magnitude changes were detected after 3 months of STZ-induced diabetes as opposed to 1 month. **B**. Set analysis of the changes at 1 month and 3 months of STZ-induced diabetes demonstrates that the vast majority of 3 month changes are not observed at 1 month of STZ-induced diabetes.

**Figure 2:**
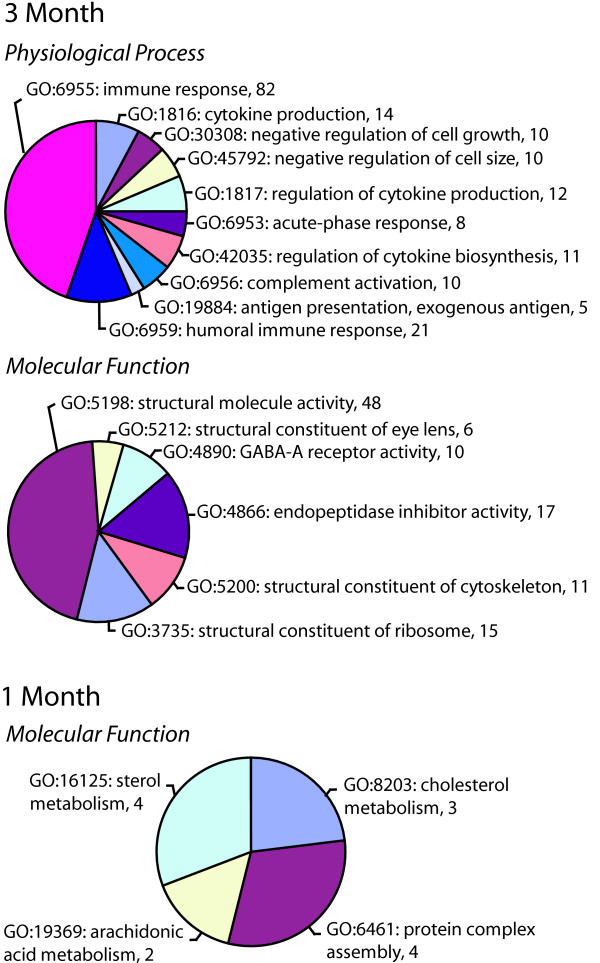
**Ontological analysis of 1 month and 3 month microarray changes**. Gene Ontology (GO) categories were queried for statistically significant overabundances of specific physiological processes or molecular functions. Statistical analysis was performed by taking the number of members of each process or function on the array and determining the number of changes that would be in that process by random chance. Processes or functions with a p value < 0.01 are given with GO accession #, function or process name and number of genes in that function or process. No statistically altered physiological processes were found for 1 month changes.

22,578 probes had detectable signal at both the 1 and 3 month time points. Expression values were generally consistent across rats and arrays in the study. Diabetes-associated changes were of a higher number and magnitude at 3 months as compared to 1 month post-STZ injection (Figure 1). The mean fold changes were 1.3 ± 0.6 fold for 1-month changes (124 genes) and 1.7 ± 0.4 fold for the 3 month changes (2310 genes). The full set of gene expression data has been deposited in the NIH/NLM Gene Expression Omnibus (GEO accession # GSE11733) [15]. To determine processes or functional groupings which are altered with diabetes, ontological analysis was performed using Gene Ontology (GO) categories [16]. Ontology results identified a number of physiological processes and molecular functions altered in the diabetic animals (Figure 2). Of special note was the significant induction of the gene ontologies for the physiological processes of cytokine production/immune response at 3 months of diabetes. Also regulated were ontologies related to cytoskeleton/crystallin proteins and GABA receptor activity.

Based on statistical analysis of microarray data and available gene information, including previous reports, 32 genes were chosen for qPCR confirmation and validation (Table 3). These 32 genes represent both genes not previously associated with DR as well as those described with DR or other retinal pathologies, including clinical reports [13]. These genes were placed *post hoc *in three categories to aid in organization and presentation. These groups are not intended to be definitive or exclusionary nor do they preclude genes from having multiple functions across the different categories and these groupings do not represent canonical gene ontology classes. Nine genes were placed in the neuronal function group, 8 in the microvascular, and 15 in the inflammatory-related group. The 32 genes examined by qPCR are only a subset of the changes observed in the microarray analysis since qPCR confirmation of microarray changes (2401 genes) was not logistically feasible. Other genes/processes which were examined further are described in other reports [17,18].

**Table 3:** Gene names, aliases, and qPCR assay numbers.

**Gene Grouping**	**Gene Symbol**	**Assay-on-Demand Catalogue #**	**Gene Name**	**Aliases**
**Microvascular**	EDN2	Rn00561135_m1	Endothelin 2	ET2
	EDNRB	Rn00569139_m1	Endothelin receptor B	ETRB, ETB
	ICAM1	Rn00564227_m1	Intercellular adhesion molecule 1	CD54
	MCT1	Rn00562332_m1	Monocarboxylate transporter 1	Slc16a1
	NPPA	Rn00561661_m1	Natriuretic peptide precursor A	ANP
	NPR3	Rn00563495_m1	Natriuretic peptide receptor 3	NPR-C, ANP-CR
	VCAM1	Rn00563627_m1	Vascular cell adhesion molecule 1	
	VEGFA	Rn00582935_m1	Vascular endothelial growth factor A	VEGF
**Inflammatory**	C1-INH	Rn01485600_m1	Complement 1-inhibitor	Serping 1, C1NH, C1l
	CCL2	Rn00580555_m1	Chemokine (C-C motif) ligand 2	MCP1, GDCF2, MCAF, SCYA2, SMCCF
	CCR5	Rn00588629_m1	Chemokine, CC Motif, Receptor 5	CCCKR5, CMKBR5, CKR5
	CD44	Rn00563924_m1	Cd44 antigen	CDW44, Pgp1
	CHI3L1	Rn01490608_m1	Chitinase 3-Like 1	GP39, YKL40
	HSPB1	Rn00583001_g1	Heat shock 27 kDa protein 1	HMN2B, HSP27, HSP28, HSP25
	JAK3	Rn00563431_m1	Janus Kinase 3	JAKL
	IL-1β	Rn00580432_m1	Interleukin 1 beta	IL-1
	IL-6	Rn00561420_m1	Interleukin 6	BSF2, HGF, HSF, IFNB2, IL-6
	LAMA5	Rn01415966_g1	Laminin, alpha 5	
	LGALS3	Rn00582910_m1	Lectin, galactoside-binding, soluble, 3	CBP35, GAL3, GALBP, MAC2
	LGALS3BP	Rn00478303_m1	lectin, galactoside-binding, soluble, 3, binding protein	MAC2BP, Serum Protein 90K
	PEDF	Rn00709999_m1	Pigment epithelium-derived growth factor	SERPINF1
	STAT3	Rn00680715_m1	Signal transducer and activator of transcription 3	APRF
	TIMP1	Rn00587558_m1	Tissue inhibitor of metalloproteinase 1	EPA
**Neuronal Function**	CHRNA4	Rn00577436_m1	Cholinergic receptor, nicotinic, alpha	BFNC, EBN, EBN1, NACRA4
	DCAMKL1	Rn00584294_m1	Doublecortin-like kinase 1	Dckl1, Cpg16
	FEZ2	Rn01468629_m1	Fasciculation and elongation zeta 2	Zygin II
	GRIN2A	Rn00561341_m1	Glutamate receptor, ionotropic, NMDA 2A	NMDAR2A, NR2A
	KCNE2	Rn02133036_s1	Isk-related voltage-gated K+ channel 2	LQT5, LQT6, MGC138292, MIRP1
	PCGF1	Rn01425394_g1	Polycomb group ring finger 1	Nspc1
	PEPT2	Rn00581522_m1	Proton-dependent high affinity oligopeptide transporter	Slc15a2
	GAT3	Rn00577664_m1	GABA transporter 3	Slc6a11
	ZNF219	Rn01414563_g1	Zinc finger protein 219	ZFP219

### qPCR confirmation and validation

Quantitative PCR analysis occurred in several steps. First, genes were confirmed for expression changes in the same set (Set 1, Table 1) of animals as used in the microarray analysis. Second, gene expression changes were validated in a second, independent set of animals (Set 2). Samples from the 3 month time point of Set 1 were exhausted before completion of the study and therefore another set (Set 3) of independent animals was generated for the 3 month time point. Genes were considered to be confirmed if the gene expression change was statistically significant at the appropriate time point in all of the animal sets tested.

**Figure 3:**
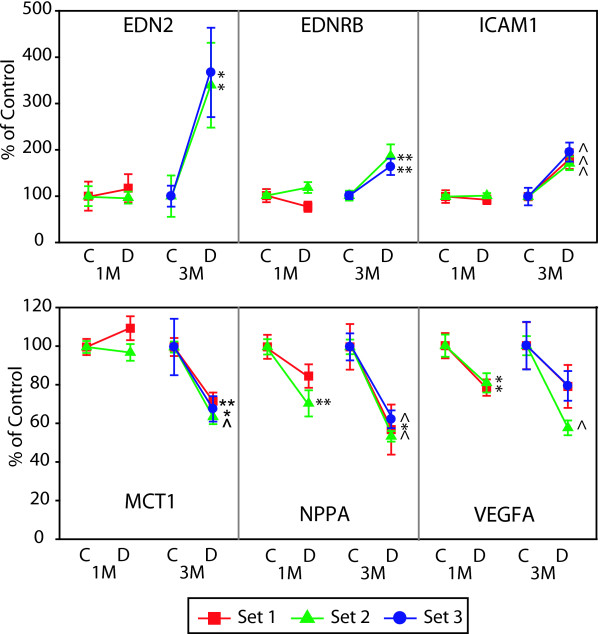
**qPCR confirmation of microvascular-related gene changes across multiple sets of animals**. qPCR data is normalized to give mean control values of 1 and the different sets are color coded per the inset. T-test, *p < 0.05, **p < 0.01, ^p < 0.001. EDN2, endothelin 2; EDNRB, endothelin receptor type B; ICAM1, intercellular adhesion molecule 1; MCT1, monocarboxylate transporter 1; NPPA, natriuretic peptide precursor type A; VEGFA, vascular endothelial growth factor A.

**Figure 4:**
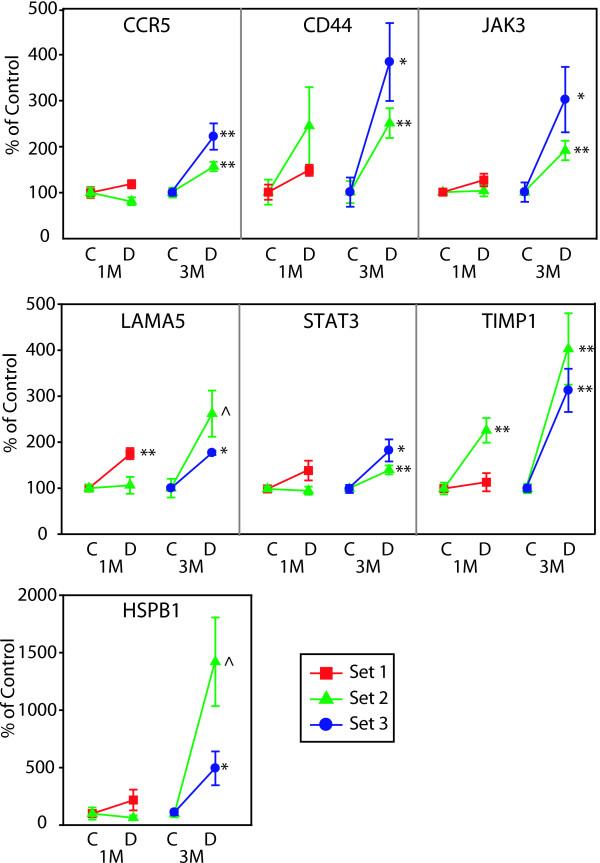
**qPCR confirmation of inflammation-related gene changes across multiple sets of animals**. qPCR data is normalized to give mean control values of 1 and the different sets are color coded per the inset. T-test, *p < 0.05, **p < 0.01, ^p < 0.001. CCR5, chemokine (C-C motif) receptor 5; CD44, cell surface glycoprotein CD44; HSPB1, heat shock 27 kDa protein 1; JAK3, janus kinase 3; LAMA5, laminin, alpha 5; STAT3, signal transducer and activator of transcription 3; TIMP1, tissue inhibitor of metalloproteinase 1.

**Figure 5:**
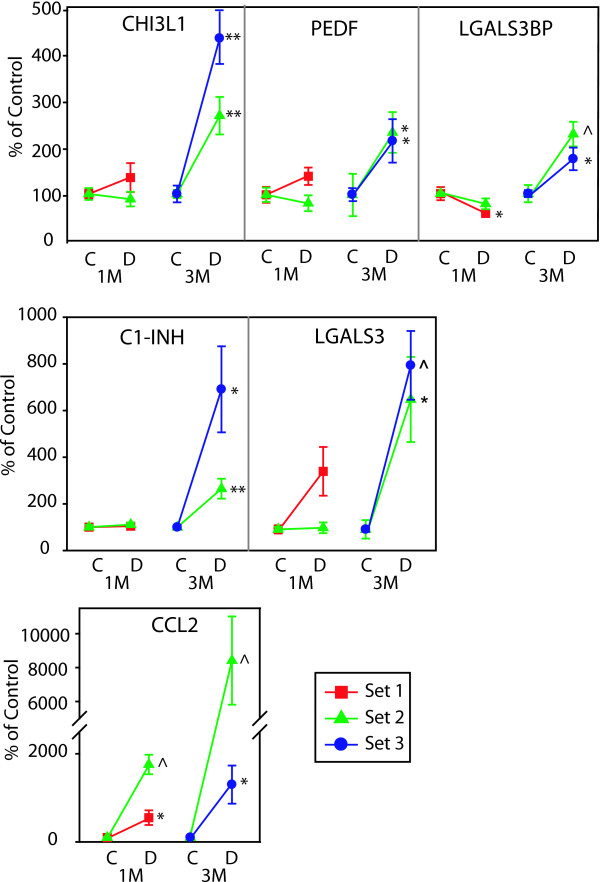
**qPCR confirmation of inflammation-related genes previously reported in patients with DR**. qPCR data is normalized to give mean control values of 1 and the different sets are color coded per the inset. T-test, *p < 0.05, **p < 0.01, ^p < 0.001. C1-INH, complement component 1 inhibitor; CCL2, chemokine (C-C motif) ligand 2; ChI3L1, chitinase 3-like 1; LGALS3, lectin, galactoside-binding, soluble, 3; LGALS3BP, lectin, galactoside-binding, soluble, 3, binding protein; PEDF, pigment epithelium-derived factor.

**Figure 6:**
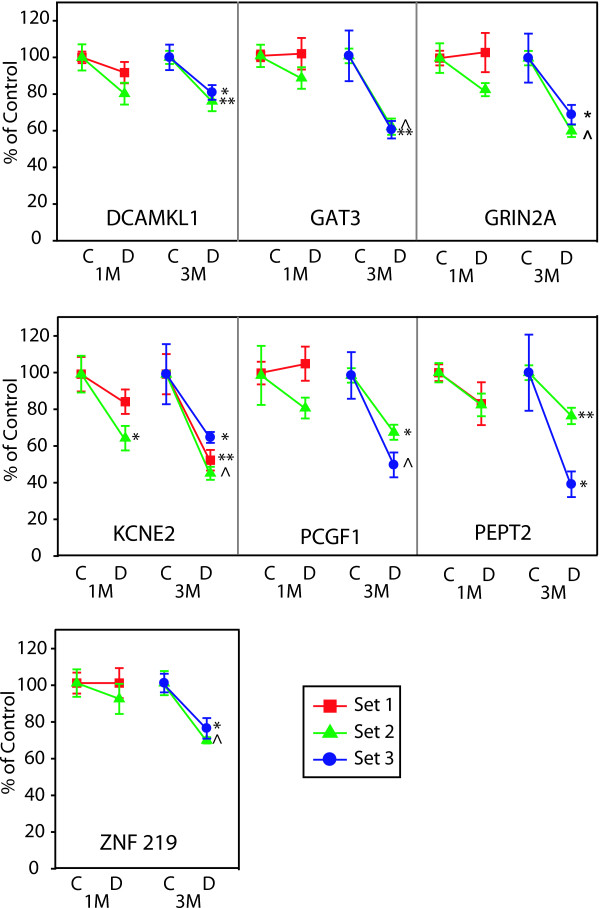
**qPCR confirmation and validation of neuronal function-related genes with consistent changes in multiple sets of animals**. qPCR data is normalized to give mean control values of 1 and the different sets are color coded per the inset. T-test, *p < 0.05, **p < 0.01, ^p < 0.001. DCAMKL1, doublecortin-like kinase 1; KCNE2, potassium voltage-gated channel, Isk-related family, member 2; PCGF1, polycomb group ring finger 1; PEPT2, proton-dependent high affinity oligopeptide transporter; GAT3, Gamma-aminobutyric transporter 3; ZNF 219, zinc finger protein 219.

Microvascular-related genes demonstrated highly reproducible changes across three independent sets of rats with 5 genes significantly altered at the 3 month time point (EDN2, EDNRB, ICAM1, MCT1, NPPA). For a complete listing of full gene names and synonyms see Table 3. VEGFA reproducibly decreased at 1 month (Figure 3). Two microvascular-related genes (NPR3, VCAM1) did not reach a statistically significant level of change in all sets of rats [See Additional file 1]. Of the 15 inflammatory genes tested, 12 genes (C1-INH, CCR5, CD44, CHI3L1, HSPB1, JAK3, LGALS3, LGALS3BP, LAMA5, PEDF, STAT3, TIMP1) were reproducibly upregulated at 3 months (Figures 4 &5). CCL2 (MCP-1) was increased at both 1 and 3 month time points (Figure 5). Of particular note were commonalities with a recent seminal report of vitreous protein levels in proliferative DR patients (Figure 5) [13]. The magnitude of induction in the inflammatory genes was much larger than other groups with inductions as high as 80 fold. Two inflammatory transcripts were not confirmed as gene expression changes [IL1β, IL6 [See Additional file 2]. All 7 confirmed neuronal function-related genes (DCAMKL1, GAT3, GRIN2A, KCNE2, PCGF1, PEPT2, ZNF219) revealed reproducible decreases at 3 months of STZ-induced diabetes (Figure 6). Two genes (CHRNA4, FEZ2) did not change in a statistically significant manner in all sets of rats [See Additional file 3]. In general, expression ratios were similar between microarray and qPCR measurements. An exact technical comparison is not possible as, in most cases, different animals (Set 1vs Set 2 or 3) and different numbers (N) of animals used in the microarray and qPCR analyses.

**Figure 7:**
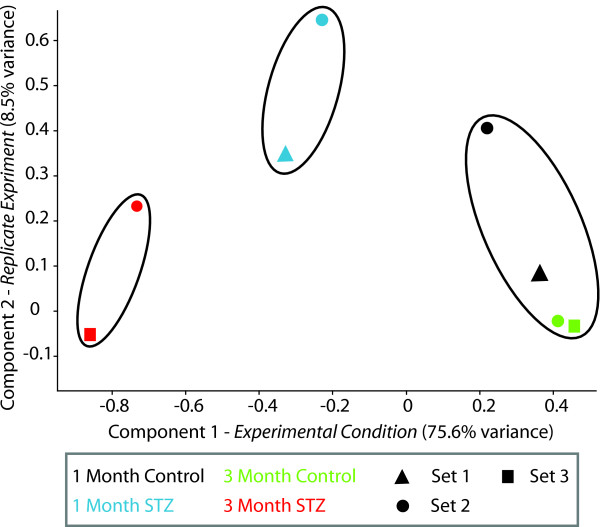
**Condition clustering using qPCR data**. qPCR data from the set of 26 validated genes in the previous figures was clustered by principle components analysis (PCA) to provide a visualization of the relationship between treatments and experiment sets. The 1st component, corresponding to the experimental treatment accounted for 75.6% of the variance, while the replicate set of animals were separated by the 2^nd ^component, accounting for 8.5% of the variance. Of note is that the 1 and 3 month STZ groups separate from the control animals and from each other, suggesting that a pattern of altered expression is beginning at 1 month. A high degree of similarity was evident between all of the control groups. Set 1 3 month data is not included because values for all genes could not be collected.

To visualize the entire pattern of gene expression measured by qPCR in the different sets of animals the qPCR gene expression data was used to generate a Principle Components Analysis (PCA) plot (Figure 7). This allows for visualization of the relationship between sets of animals at different time points and treatments. The first component provides separation between control and diabetic sets and accounts for 75.6% of the study variance providing further evidence of the reproducibility of the diabetic effect. Furthermore, the 1 month animal sets represent a midpoint between control and 3 month diabetic animals. This demonstrates that while most of these genes are not statistically altered at 1 month, there is a gene expression pattern developing prior to onset of significant increases in vascular permeability and apoptosis. The second component accounted for a much smaller percentage of the study variance (8.5%) and represents the variability between the independent sets of animals. Therefore, these gene expression changes are a robust effect compared to inter-experiment variability.

## Discussion

The purposes of this study were to examine retinal permeability and apoptosis at 1 and 3 months of diabetes and to identify accompanying retinal mRNA expression changes. The time points of this study were chosen as models of the chronic progressive nature of complications such as DR and are consistent with previous studies [14,19]. The findings of the study demonstrate that changes in retinal expression of inflammatory, microvascular, and neuronal transcripts occur in concert with functional changes in retinal permeability and apoptosis.

The increased vascular permeability reported here (52%) closely replicates our previous findings of increased vascular permeability (62%) at 3 months of diabetes [19]. Similarly, we confirmed our previous finding of increased caspase-3 activity at 3 months of diabetes [14]. We both confirm that these changes are evident at 3 months of diabetes and report that there are no changes in vascular permeability and caspase-3 activity at 1 month by these methods. Lack of significant change in permeability and apoptosis with the assays used at 1 month of diabetes provides the opportunity to assess those gene expression changes which occur before and/or concomitantly with functional alterations.

Thirty two genes significantly altered in the microarray analysis were subjected to qPCR confirmation. After qPCR confirmation of 26 of these genes in multiple sets of animals, these genes were placed in the microvascular, neuronal function and inflammatory gene sets because they represent central hypotheses of retinal cellular alterations with diabetes. While the interactions of the microvascular and neuronal cells along with inflammatory activation are not fully understood, the various retinal cells clearly function in a coordinated fashion with integrated metabolism and cell – cell contacts. We have previously postulated a "feed-forward" interaction concept in which defects in vascular, neural and inflammatory responses to diabetes perpetuate the neurovascular degeneration [9]. These genes were tested across replicate sets of rats to determine those which were reproducibly altered. A major finding of this work is the reproducibility of these changes across independent experiments. Many more differentially regulated genes from the microarray analysis remain to be confirmed and expanded upon [as described elsewhere [17,18]]. As mRNA levels do not always correspond to protein expression levels, future studies will be needed to address changes in protein levels and precise cellular localization of these changes.

### Microvascular

Microvascular dysfunction is a hallmark of DR, with numerous reports of diabetes-induced changes in humans as well as in animal models. Several reproducibly altered microvascular transcripts were confirmed in this study (Figure 3). ICAM1 (intercellular adhesion molecule 1) is commonly associated with retinal disease states, as adhesion molecules are produced by vascular endothelial cells are induced during the proliferative stages of DR [20]. ICAM1 suppression decreases leukostasis and vascular leakage in retinas of STZ-diabetic rats [21] and ICAM1 deficient STZ treated mice demonstrate reduced leukocyte adhesion and vascular permeability as compared to wild-type [22].

While the EDN1 (endothelin 1) alterations have been well described in DR, induction of EDN2 (endothelin 2), a potent vasoconstrictor, and the endothelin receptor EDNRB (endothelin receptor B) have been described after 6 months of diabetes and light injury [23,24]. NPPA (natriuretic peptide precursor type A; also known as atrial natriuretic peptide, ANP) is an antigrowth factor in endothelial cells that counteracts the angiogenic and permeability actions of VEGF. NPPA is also protective against NMDA-induced retinal neurotoxicity [25]. Our findings concur with a recent report of decreased NPPA mRNA and protein levels at 3 months but not 1 month of STZ-diabetes in rats [26].

Retinal mRNA levels of MCT1 (monocarboxylic acid transporter 1), transporter of lactate and pyruvate between astrocytes and neurons, were previously reported to be unchanged in the rat retina at 10 weeks of STZ-induced diabetes [27]. However, MCT1 mRNA levels were consistently decreased at 3 months of diabetes in this study.

Induction of retinal VEGFA (vascular endothelial growth factor) protein with diabetes is well known, but previous studies of VEGF mRNA have produced conflicting results. For example, retinal VEGF mRNA levels have been reported to be unchanged at 3 months [28], increased at 6 months [29], and decreased at 6 months [12] duration of STZ-induced diabetes in the rat. We demonstrate a consistent decrease in VEGF mRNA expression in multiple independent sets of animals. The lack of concordance with previously described increases in VEGF protein suggests a post-translational mechanism for VEGF protein regulation. In total, these microvascular gene expression differences indicate a synergistic increase in microvascular dysfunction (ICAM1, EDN2, and EDNRB) and decrease in a potential compensatory mechanism (NPPA).

### Inflammation

Altered inflammatory equilibrium has the potential to exacerbate or be induced by both the neurodegenerative and microvascular dysfunctions typical of DR [9]. Clinically, elevated vitreal levels of pro-inflammatory cytokines have been described (e.g. [10,13]). Our findings confirm the induction of many of these same factors in a rodent model and provide evidence of a number of novel inflammation-related changes (Figures 4 &5), some of which have also been described in Müller cells after 6 months of experimental diabetes [12]. The majority of the genes can be described as pro-inflammatory (CCL2, CCR5, CD44, CHI3L1, JAK3, STAT3, TIMP1, LAMA5, LGALS3, and LGALS3BP) with some potentially anti-inflammatory changes (PEDF, HSPB1, C1-INH).

Pro-inflammatory changes included chemokines, receptors, and signaling molecules. All of these genes have multiple points of interaction with inflammatory and chemotactic cascades which themselves are often associated with breakdown of vascular barrier properties and neurodegeneration. Specifically, elevated CCL2 (chemokine, CC motif, ligand 2), (also known as Scya2; Small inducible Cytokine A2, and MCP-1; monocyte chemotactic protein 1) has been reported in the vitreous of patients with proliferative DR [30], with increased CCL2 correlating with increasing severity of proliferative DR [31]. The high level of induction at 1 month (20.9 fold in set 1 and 5.4 fold in set 2) and even higher elevation at 3 months (84.1 fold in set 2 and 13.0 fold in set 3) demonstrates for the first time that CCL2 mRNA is increased in the retina of STZ-diabetic rats and suggests a very strong pro-inflammatory response that occurs within one month of diabetes and progresses with time.

CCR5 (chemokine, CC motif, receptor 5) a RANTES receptor facilitates leukocyte infiltration into the retina [32] and could work synergistically with elevated levels of RANTES present with diabetes [33]. CD44 is a widely expressed glycoprotein that plays a direct role in leukocyte trafficking to the retina [34]. The systemic plasma levels of the primary ligand for CD44, hyaluronan, are increased in diabetic patients [35]. In a manner similar to CCR5, increased CD44 levels could work synergistically with elevated levels of its ligand to recruit leukocytes.

JAK3 (janus kinase 3) expression requires cytokine signaling and inhibition of JAK3 prevents STAT3 activation [36]. STAT3 (signal tranducer and activator of transcription 3) has been localized to the inner nuclear layer and ganglion cells [37]. TIMP-1 (tissue inhibitor of metallopeptidase1) expression is regulated through STAT3 [38] and TIMP-1 is induced in the epiretinal membranes of Type 1 and Type 2 diabetic patients [39].

CHI3L1 (chitinase 3-like 1) is an inflammatory marker associated with acute and chronic inflammation, chemotaxis, and angiogenesis. CHI3L1 plasma levels in Type II diabetes patients are positively correlated with insulin resistance and endothelial dysfunction [40]. LGALS3 (lectin, galactosidase-binding, soluble, 3), also known as GAL3 (galectin 3), plays many roles in inflammatory responses as an advanced glycation end-products (AGE) receptor [41]. AGEs are elevated in diabetic tissues and sera [42]. Removal of LGALS3 inhibits AGE-mediated retinal ischemia and suggests an important role for LGALS3 in AGE-related pathophysiology [43]. LGALS3BP (lectin, galactosidase-binding, soluble, 3, binding protein), is a cell adhesion binding protein for LGALS3 and is elevated in patients with DR [13]. There are no reported data describing the role of LAMA5 (Laminin, alpha-5) retina, however, laminin-LGALS3 interactions can promote leukocyte adhesion [44].

The three potentially anti-inflammatory gene expression changes confirmed (C1-INH, HSPB1, and PEDF) may represent a compensatory response to aspects of the pro-inflammatory response. C1-INH (complement component 1 inhibitor), also known as Serping1 [serine (or cysteine) peptidase inhibitor, clade G, member1], protein expression is increased in DR patients and C1-INH blocks the increased permeability caused by vitreal injection of carbonic anhydrase [13]. HSPB1 (heat-shock 27-kD protein 1), also commonly known as Hsp27, inhibitis both caspase-dependent and -independent apoptosis [45] and is protective against oxidative stress breakdown of barrier integrity [46].

The observation that PEDF (pigment epithelium-derived factor) counteracts the effects of VEGF through antiangiogenic properties [47] has been challenged by recent report of elevated levels of PEDF in patients with proliferative DR than controls [48]. In fact, increased vitreal levels of PEDF have been described in other DR clinical reports [13]. We also observe an induction of PEDF and more studies will be needed to determine if PEDF induction is a compensatory response to other inflammatory and angiogenic changes or if it has a different purpose.

The majority of these inflammatory changes occur at 3 months of diabetes, the same time as the functional changes. However, the marked induction of CCL2 occurs before observed changes in permeability and apoptosis. This may represent an initial event which leads to the cascade of events eventually resulting in retinal dysfunction. Examination of the entire pattern of gene expression (Figure 7) raises the possibility that small magnitude changes could be working in concert to progressively cause later retinal dysfunction.

### Neuronal function

We have previously reviewed the neuronal components of DR pathophysiology and the role of neurodegeneration in vision loss [9]. The data presented in this study establish transcriptional alterations in a set of genes related to neuronal function, all suggestive of neurodegeneration. DCAMKL1 (doublecortin-like kinase 1) is highly expressed in the ganglion cell layer [49] and functions with Doublecortin in microtubule stabilization. Further studies will be needed to determine if a decrease in DCAMKL1 could be associated with reductions or weakening of synapses observed in diabetic rat retinas [17].

PEPT2 (proton-coupled peptide transporter, Slc15a2) transports peptides across membranes in a low capacity/high affinity manner and is expressed in the inner nuclear layer Müller cells and in astrocytes of the retinal ganglion layer [50]. GAT3 (GABA transporter 3, Slc6a11) is found on both neuronal and glial cells in the retina [51]. The possible role of decreased GABA uptake in retinal pathology is unclear, but could induce electrophysiological changes in the diabetic retina [52].

KCNE2 (isk-related voltage-gated K+ channel 2) colocalizes with KCNQ channel members to regulate channel activity. While KCNE2 is uncharacterized functionally in the retina, it is highly enriched in rods [53]. GRIN2A (ionotropic glutamate receptor 2a, NMDAR2a) receptors are poorly characterized in the retina, but could play a role in the altered dark adaptation as neurotransmission is increased in the dark [54].

PCGF1 (polycomb group ring finger 1), also known as nervous system polycomb 1 (NSPc1), is a nervous system transcription repressor regulated by PKC signaling [55]. ZNF219 (zinc finger protein 219, or Zfp219) is an uncharacterized transcriptional repressor [56] with highest expression in the brain and nervous system.

While existing knowledge of these neuronal function-related genes in the retina is limited, the collective decreases observed indicate a compromised neuronal phenotype. Current work suggests that neurodegeneration in diabetic retinopathy includes a synaptic component that may lead to reduced neuronal function and signaling [17] further developing the concept of a compromised neuronal phenotype [9].

Lastly, to visualize the interactions between the different genes confirmed by qPCR, the Ingenuity Pathway Analysis (IPA) system was used to generate a network of gene interactions. Twenty-two of the genes confirmed by qPCR, along with increased caspase activity and decreased insulin levels created a network of interactions [See Additional file 4]. The additional genes and vascular permeability values were not able to be included in a rigorously generated network. This does not preclude them from having roles in the network. While these network analyses can only use known interactions, these increases in expression or activity (red fills) and decreases in expression or levels (green fills) could act synergistically, as we have previously proposed [9]. Furthermore, when previously described roles of these transcripts in functions and diseases (inflammation, diabetes, non-proliferative DR, and neuropathy) are overlaid onto the network, a number of interactions are obvious (orange highlights). As we have discussed above, the combination of these changes could produce a neurovascular inflammatory response.

## Conclusion

Each of the genes described has potential roles in the increased vascular permeability and apoptosis observed. A critical finding of this study is that a number of inflammatory genes (C1-INH, CCL2, LGALS3, LGALS3BP, and PEDF) are induced in a manner similar to clinical reports of patients with DR [13]. These inductions occur in tandem with increases in vascular permeability and apoptosis that are analogous to the clinically observed changes in barrier properties and apoptosis. These findings suggest that the STZ-rat could be used to model aspects of clinical DR, especially inflammation. The STZ-rat model may provide a useful preclinical tool to investigate the specific roles of these genes in diabetes-induced retinal pathophysiology. This model may also serve as a preclinical model for studies of potential therapeutics to assess their efficacy in preventing or reversing deregulation of retinal gene expression and pathophysiology with diabetes.

The individual genes identified and confirmed here are points of interest for future studies focused on elucidating the role of each gene product in normal retinal physiology and DR. Together, the panel of genes altered with diabetes provides further evidence to support the hypothesis of a feed-forward cycle of chronic inflammation, neurodegeneration, and compromised blood-retinal barrier function in the pathophysiological development of DR.

## Methods

### Animals

Experiments were performed following the ARVO Statement for the Use of Animals in Ophthalmic and Vision Research protocols. All rats were maintained by the Penn State JDRF Animal Core in accordance with the Institutional Animal Care and Use Committee guidelines under specific pathogen-free conditions and monitored by quarterly sentinel testing. Sprague Dawley male rats (Charles River Laboratories, Wilmington, MA) arrived at 100–125 grams. After one week and following an overnight fast, diabetes was induced by intraperitoneal injection of 65 mg/kg streptozotocin (Sigma-Aldrich, St. Louis, MO) in 10 mM sodium citrate pH 4.5 vehicle. Control rats were injected with an equal dose of vehicle only. Rats had free access to food and water, and were on a 12 hour light/dark cycle. Blood glucose level and body weight were measured 6 days post-STZ or vehicle injection, and biweekly throughout the experiment. Only rats with blood glucose levels >250 mg/dL at the time of the original test were included in the diabetic groups (Table 1, Online Appendix). No exogenous insulin was delivered. At the time of retinal harvest, rats were given a lethal dose of pentobarbital, 100 mg/kg, (Ovation Pharmaceuticals Inc., Deerfield, IL) by intraperitoneal injection. Retinas were excised and quickly frozen in liquid nitrogen.

### Vascular permeability

Blood-retinal barrier permeability was measured in rats after 1 and 3 months after streptozotocin (STZ) treatment using a FITC-BSA modification of the method described by Xu et al. [57] using FITC-BSA. Under ketamine/xylazine anesthesia (67/6.7 mg/kg body weight, i.m.), animals received femoral vein injection of 100 mg/kg body weight of fluorescein isothiocyanate-bovine serum albumin (FITC-BSA) in sterile PBS (Sigma, St. Louis, MO). After 2 hours, the animals were anesthetized again with ketamine/xylazine. The abdominal cavity was opened and 1 ml blood was drawn from the inferior vena cava to obtain the FITC-BSA concentration in plasma. Subsequently, the heart of the animal was perfused for 2 min with citrate buffer (50 mM, pH 3.5, 37°C) containing 1% paraformaldehyde (Fisher, Pittsburgh, PA). After perfusion, retinas were harvested from both eyes and dried in a Savant Speed-Vac (Thermo Scientific, Waltham, MA). The FITC-BSA was extracted by incubating each retina in 200 μl PBS with 1% Triton X-100 (Sigma) and 0.1% sodium azide (Fisher) rocking at room temperature overnight. The extract was transferred to a filter tube and centrifuged at 5,000 × g for 3 hours. Plasma and retina extract samples were assayed in triplicate of 50 μl/well in a 96-well black/clear bottom plate (BD Biosciences, Franklin Lakes, NJ) for fluorescence with a SpectraMax Gemini EM fluorescent plate reader (Molecular Devices, Sunnyvale, CA) based on standard curves of FITC-BSA with excitation at 488 nm and emission at 520 nm. The auto-fluorescence background was subtracted with the retina obtained from the rat without FITC-BSA injection. The retinal permeability was calculated and expressed in μl plasma/g dry retina weight/hr circulation.

### Caspase activity

Caspase-3 activity was measured in retinal protein homogenates using the fluorometric CaspACE assay system (Promega). Rats were anesthetized with 100 mg/kg sodium pentobarbital. Retinas were excised, and placed in 60 μL of cold lysis buffer (25 mM Hepes, pH7.5, 5 mM MgCl_2_, 5 mM EDTA, 5 mM DTT, 2 mM PMSF, 10 μg/mL leupeptin, 1% NP40). The retina was gently sonicated followed by 30 minute incubation at 4°C and 20 min centrifugation at 16,000 g at 4°C. Caspase-3 activity was measured in the supernatants using the fluorometric CaspACE assay system (Promega) and protein content was measured by BioRad protein assay. CaspACE assay was performed according to the manufacturer's protocol using 50 μg retinal protein and 37°C incubations in a 96-well plate format.

### Microarray analysis

Microarray analysis was performed in the Penn State College of Medicine Functional Genomics Core Facility according to standard procedures.

Total RNA was isolated with Tri-Reagent/BCP (Molecular Research Center, Cincinnati, OH) following standard methods [58] and quality and quantity was assessed using the RNA 6000 Nano LabChip with an Agilent 2100 Expert Bioanalyzer (Agilent, Palo Alto, CA).

1 μg RNA was transcribed to cRNA following the protocol of the Codelink iExpress cRNA Prep & Hyb Kit (GE Healthcare). After second strand synthesis and purification of dsDNA using Qiaquick spin columns (Qiagen, Valencia, CA), T7 reaction buffer, NTPs, 10 mM biotin-11-UTP, and T7 polymerase were added to the dsDNA and the reaction was incubated at 37°C for 14 hours. Biotin labeled cRNA was then purified using RNEasy columns (Qiagen) followed by quantitation. 10 μg from each sample was fragmented, denatured and then hybridized to Codelink rat whole genome microarray slides for 18 hours at 37°C. Slides were then incubated at room temperature with Alexa Fluor 647 labeled steptavidin for 30 minutes followed by washing.

Microarrays were scanned with an Axon 4000B scanner with GenePix4 v4.0 software at a 5 μm resolution at 635 nm with laser power at 100%, PMT voltage at 600 V, focus position 0 μm, and lines to average = 1. Images were then imported into CodeLink Expression Analysis Software v4.1 (GE Healthcare). Initial quality control (positive and negative controls), exclusion of manufacturing defects (MSR spots), background subtraction, and intra-array normalization was then performed and the results exported to GeneSpring GX 7.3 (Agilent Technologies).

For data analysis, data was imported into GeneSpring GX 7.3 (Agilent Technologies) and signal values less than 0.01 were set to 0.01, arrays were normalized to the 50^th ^percentile, and individual genes normalized to the median. Values were then normalized on a per gene basis to the control group for each of the two time points (1 or 3 Month). Potential differential expression was determined with a one-way ANOVA (variances not assumed to be equal), p < 0.01 and filtered for 1.2 fold and greater differences in expression in accordance with standards for microarray analysis [59,60]. The use of a combination of statistical and fold-change cutoffs as opposed to traditional multiple testing corrections (*e.g*. Bonferroni) produce gene lists with the lowest rate of type I and type II errors. 1.2 fold was chosen as the fold change cutoff, as this magnitude change is at the lower range of changes confirmable by qPCR. Lastly, probe sequences on the array were searched against rat genome sequences to eliminate any probes for sequences removed by standard genome processing and annotation. The full complement of microarray data has been deposited in the NIH/NLM Gene Expression Omnibus (GEO accession # GSE11733) [15].

### Ontological analysis

Ontological analysis used Gene Ontology (GO) categories [61]. Differentially expressed processes or functional categories were determined statistically, similarly to a previously described approach [16] using GeneSpring GX software. This analysis determined the number of genes in a category present on the array and the number of expression changes that would be part of that category by random chance given the number of differentially expressed genes.

### Pathway and network analysis

Ingenuity Pathway Analysis (Ingenuity Systems, Redwood City CA) was used to create a network from the qPCR confirmed gene expression results. Insulin levels, caspase activity, and vascular permeability data were also included.

### Clustering and statistical analyses

For statistical analysis of qPCR data, standard parametric t-tests (α < 0.05, two-tailed) were used. Each set (1 and 3 month) of animals was treated independently since the animals were generated and sacrificed separately. Principal components analysis clustering was performed using a mean centered and scaled algorithm (GeneSpring 7.3, Agilent Technologies).

### Quantitative RT-PCR

Quantitative PCR analysis was performed using the 7900 HT Sequence Detection System (Applied Biosystems, Foster City, CA), 384-well optical plates, and Assay-On-Demand (Applied Biosystems) gene specific primers and probes. SDS 2.2.2 software and the 2^-ΔΔCtCt ^analysis method [62] were used to quantitate relative amounts of product using β-actin as an endogenous control. β-actin levels were determined to be unchanged in an absolute quantitation experiment (data not shown). For a full listing of primer/probe sets see Table 2, Online Appendix. For statistical analysis of qPCR data, standard parametric t-tests (α < 0.05, two-tailed) were used. Each set (1 and 3 month) of animals was treated independently since the animals were generated and sacrificed separately.

## Authors' contributions

RMB, KMP, and HDVB carried out the microarray and qPCR assays, HDVG and AJB generated samples and contributed to the writing of the manuscript, DAA and C-ML performed the vascular permeability studies, KFL performed the caspase assays, TWG contributed to the experimental design and manuscript preparation, SKB directed the generation of samples, and WMF performed the bioinformatic analyses and prepared the manuscript. All authors read and approved the final manuscript.

## Pre-publication history

The pre-publication history for this paper can be accessed here:



## Supplementary Material

Additional file 1 Microvascular changes not validated by qPCR. Click here for file

Additional file 2 Inflammatory changes not validated by qPCR. Click here for file

Additional file 3 Neuronal changes not validated by qPCR. Click here for file

Additional file 4 Network analysis of confirmed changes. Click here for file
