# Supplementary material for: Whole genome assessment of the retinal response to diabetes reveals a progressive neurovascular inflammatory response
Source: BMC Med Genomics. 2008 Jun 13;1:26. doi: 10.1186/1755-8794-1-26 (PMC2442612; doi:10.1186/1755-8794-1-26)
Supplement: Additional file 2 — Inflammatory changes not validated by qPCR. [file 1755-8794-1-26-S2.pdf]

Inflammatory Supplement

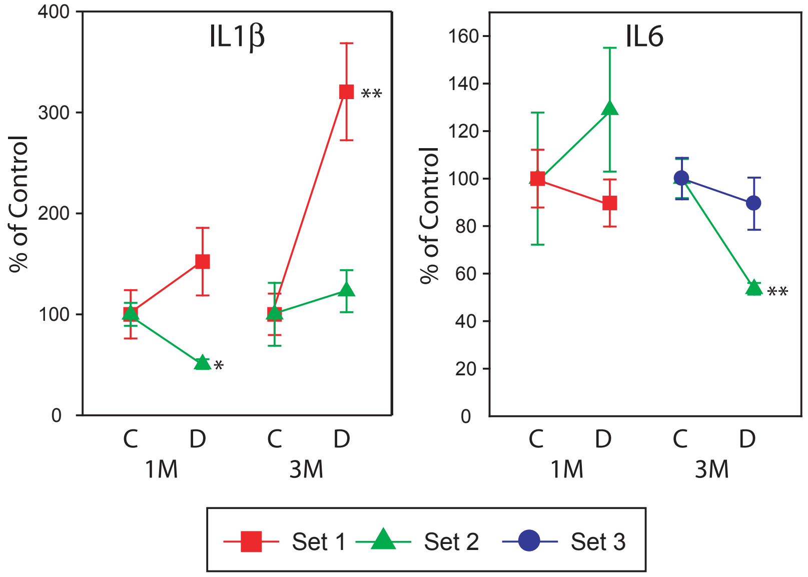

Supplemental Figure 2: Inflammatory changes not validated by qPCR. qPCR data is normalized to give mean control values of 1 and the different sets are color coded per the inset. T-test, **p<0.01, . IL1β, interleukin 1, beta; IL6, interleukin 6.
