# Supplementary material for: Whole genome assessment of the retinal response to diabetes reveals a progressive neurovascular inflammatory response
Source: BMC Med Genomics. 2008 Jun 13;1:26. doi: 10.1186/1755-8794-1-26 (PMC2442612; doi:10.1186/1755-8794-1-26)
Supplement: Additional file 3 — Neuronal changes not validated by qPCR. [file 1755-8794-1-26-S3.pdf]

Neuronal Function Supplement

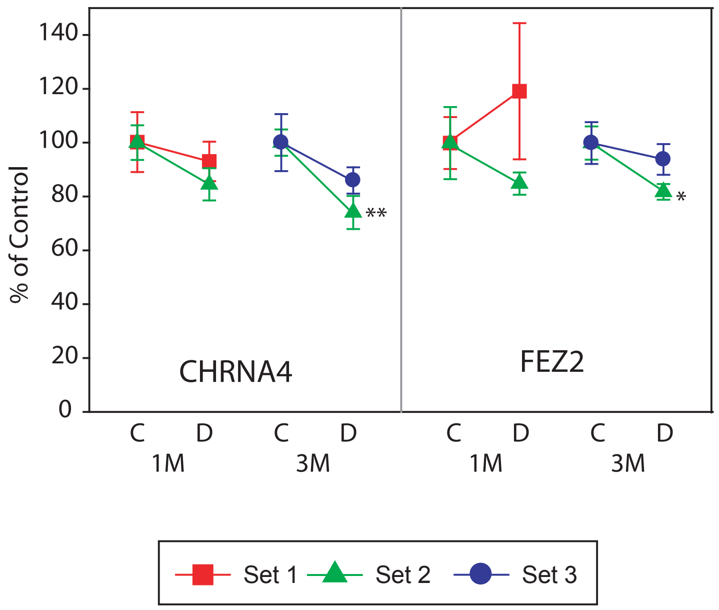


**Supplemental Figure 3: Neuronal function changes not validated by qPCR.** qPCR data is normalized to give mean control values of 1 and the different sets are color coded per the inset. T-test, *p<0.05, **p<0.01, ^p<0.001. CHRNA4, cholinergic receptor, nicotinic, alpha 4; FEZ2, fasciculation and elongation protein zeta 2.
