# Supplementary figures and images for: Whole genome assessment of the retinal response to diabetes reveals a progressive neurovascular inflammatory response
Source: BMC Med Genomics. 2008 Jun 13;1:26. doi: 10.1186/1755-8794-1-26 (PMC2442612; doi:10.1186/1755-8794-1-26)

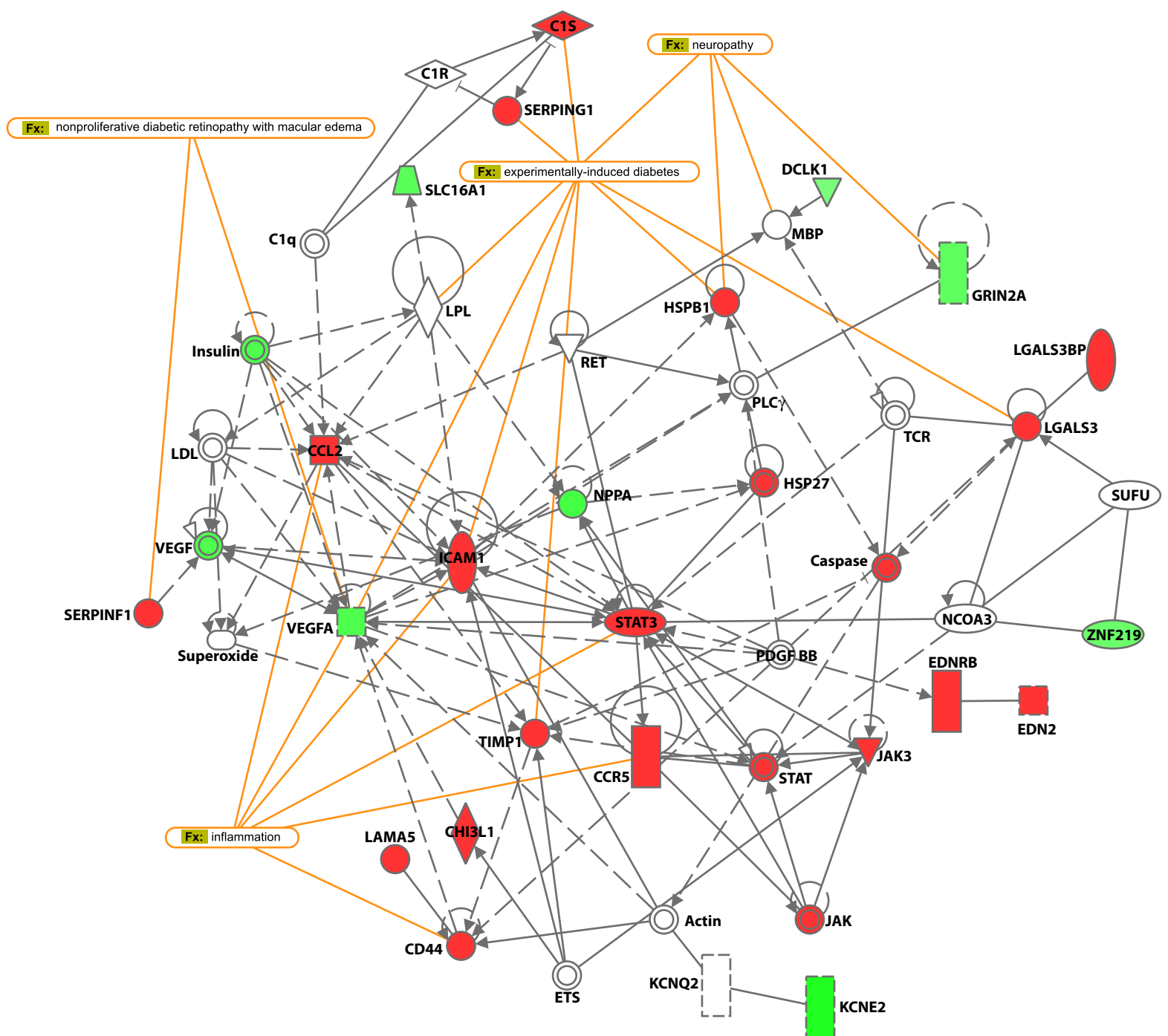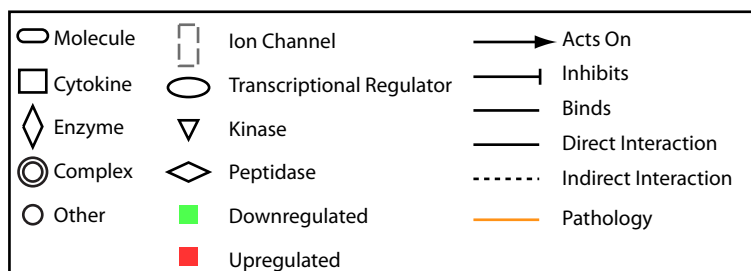

Supplement: Additional file 4 — Network analysis of confirmed changes. [file 1755-8794-1-26-S4.pdf]
